# The impact of positive psychological intervention on college students' mental health and behavioral performance: the moderating role of teacher empathic concern

**DOI:** 10.3389/fpsyg.2025.1650042

**Published:** 2025-11-10

**Authors:** Hui Wang, Lei Xi

**Affiliations:** School of Management, Anhui Science and Technology University, Bengbu, China

**Keywords:** subjective wellbeing, physical exercise, mental health, behavioral performance, teacher empathic concern

## Abstract

**Introduction:**

College students‘ mental health and behavioral performance have been a hot topic of concern among scholars. With the heavy academic pressure, high social expectations, and uncertainty about future career development, college students' anxiety and depression aspects have become more and more prominent. However, the relationship between positive heart interventions (subjective wellbeing and physical exercise), teachers‘ empathetic attention, college students' mental health and behavioral performance, and their mechanisms still need to be further studied.

**Methods:**

This study aimed to investigate the relationship between positive mindfulness interventions and college students' mental health and behavioral performance, and to verify the moderating role of teachers' empathetic attention between the two. In addition, this study examined the current status of positive mindfulness interventions, teachers' empathic concern, mental health and behavioral performance of 260 Chinese university students by means of a questionnaire survey.

**Results:**

The results of the study showed that positive heart interventions, subjective wellbeing, and physical activity had a significant role in promoting college students' mental health and behavioral performance, positive heart interventions (β = 0.488, *P* < 0.001), (β = 0.474, *P* < 0.001) subjective wellbeing (β = 0.327, *P* < 0.001), (β = 0.362, *P* < 0.001), and physical activity (β = 0.254, *P* < 0.001), (β = 0.203, *P* < 0.001). In addition, teachers' empathetic attention played a positive moderating role between positive heart interventions and college students' mental health (β = 0.332, *P* < 0.001). Teachers' empathic concern played a positive moderating role between positive heart intervention and college students' behavioral performance (β = 0.223, *P* < 0.001).

**Conclusion:**

This study provides valuable suggestions for teachers, doctors, and parents to better safeguard the state of mental health of college students and to regulate the behavioral performance of college students to be consistent with the ideal goals they pursue.

## 1 Introduction

In today's competitive social environment, young adults in higher education are often faced with tremendous pressure caused by heavy academic pressure, career confusion, and complex social relationships (Li and Gao, 2024; Zhou and Guo, 2023; Dessauvagie et al., 2022), and these mixed potential factors can negatively affect college students‘ mental health and behavior, and may cause serious mental health problems such as mood and anxiety disorders. negatively, and may also cause serious mental health problems such as anxiety and depression (Lee et al., 2023). The transitional phase between childhood and adulthood, adolescence holds immense significance, represents a pivotal period for self-worth formation and individual physical and mental growth, and students are very prone to heart problems confronted with excessive anticipations from family, school, and society (Wang and Fan, 2023). Adolescents suffering from heart health problems are prone to develop emotional and behavioral disorders (Auerbach et al., 2018), eating disorders (O'neil et al., 2014). In view of the complexity of college students' mental health and behavioral manifestations, in-depth research on this not only can prevent the negative impacts of college students‘ growth process, promote students' physical, mental and academic health and progress, but also have a far-reaching impact on their future career development.

As people's standard of living and quality of life improve, the pursuit of spirituality has become more prominent, making subjective wellbeing an important indicator of quality of life (Skevington and Böhnke, 2018). Subjective wellbeing embodies an important theme of positive psychology, including an individual's attitudinal and emotional assessment of life (Csikszentmihalyi and Larson, 2014). Established research has found that individuals who master subjective wellbeing are more value-creative and have social attributes (Diener, 1984). When students‘ subjective wellbeing increases, students may be happier and tend to look toward the future and live positively (Dalmiş et al., 2025). Adolescence is a critical period for the progression of subjective wellbeing (Froh et al., 2009), but the level of subjective wellbeing in adolescents is relatively low, and developing and nurturing subjective wellbeing is a difficult task (Patton et al., 2016). Given that subjective wellbeing is a highly effective positive heart intervention as well as its role in college students' mental health and behavioral performance, the present study supplies insight into the heart mechanisms of subjective wellbeing among college students.

As an active lifestyle, physical activity has received widespread focus in the realm of adolescent mental health, and physical activity not only enhances physical fitness, but also contributes to the improvement of adolescents‘ mental status and reduces the occurrence of depression (Herbert, 2022). In China, despite the popularization of physical activity, adolescents' high academic pressure, insufficient time for physical activity, and lack of sports resources have led to a low participation rate (Zhang et al., 2022). Previous research has demonstrated that strengthening bodily movement can improve the ability to adapt to the natural and social environments (Yan et al., 2020), enhance the spirit of teamwork (Xia and Shi, 2025), and improve interpersonal relationships (Zhong et al., 2024). However, in reality, college students are anxious about social competition, academic pressure, and the future, which makes the role of physical exercise to relieve anxiety in their spare time ineffective (Wang et al., 2023). Therefore, how physical activity, as a positive mental intervention, affects the mental health and behavioral performance of college students still needs further analysis.

In education, teaching is not merely the traditional dissemination of knowledge. Teachers must have emotional engagement, understanding and concern for students' viewpoints and emotions (Aldrup et al., 2022). Emotional empathy is a series of active processes in which people experience shared emotional responses with others (Bernhardt and Singer, 2012). Recent research emphasizes that when students encounter learning difficulties, teachers' empathetic abilities can help them overcome the difficulties and enhance their own professional skills and resilience (Zhu et al., 2019; Wink et al., 2021). Understanding-oriented teachers improve students' psychological states by providing emotional support to alleviate their learning pressure and anxiety (Derakhshan et al., 2022). Given that teacher empathy focuses on the importance of the teaching process, it is necessary to incorporate teacher empathy focus into the factors influencing the mental health and behavioral performance of college students.

## 2 Literature review and research hypothesis

### 2.1 Positive heart interventions and mental health

Subjective wellbeing involves a person's cognitive judgments and emotional responses to life, and is an important reference indicator for measuring the state of adolescents' mental health (Diener, 2000; Lu et al., 2024). According to Tov (2018), supervisory wellbeing mainly consists of regularized feelings of happiness, rare feelings of dissatisfaction, and overall evaluation of feelings about the current situation in life. The research of Diener and Seligman (2004) shows that if people have a high level of subjective wellbeing, they will often be accompanied by positive emotions, they will become happier and more hopeful about the future, and they can cope better with external pressures and maintain a healthy state of mind. The theory of heart capital suggests that individuals with more heart capital can reduce the negative impact of negative factors on physical and mental health (Sabaityte, 2014), and subjective wellbeing, as a positive heart resource, plays an irreplaceable role in the heart health of adolescents (He et al., 2023). Students' subjective wellbeing is derived from the positive impact of quality life satisfaction, which not only increases students' positive emotions, but also reduces tension, uneasiness and anxiety about the future, thus safeguarding physical and mental health (Dalmiş et al., 2025).

The World Health Organization (World Health Organization, 2020) states that physical activity depends mainly on physiological and psychosocial mechanisms to guarantee the level of mental health. Physical activity is widely recognized by society at large for having many advantages that greatly enhance mental health and heart resilience (Sun et al., 2019). Regular participation in physical activity can help adolescents with weight management, ensure cardiovascular fitness, drive academic achievement, and maintain a good level of mental health (Hills et al., 2015). Given young people's lack of time for exercise, physical activity with intensity can reduce symptoms of anxiety and depression, enhance intrinsic motivation to participate in sports, improve cognitive levels, and positively impact adolescents‘ mental health (Alves et al., 2021). Regular participation in physical activity improves body image, increases self-esteem and enhances self-identity, reduces individuals' loneliness, and provides individuals with a feeling of emotional connectedness and social backing, which helps individuals to live a normal, happy life and maintain a positive state of mental health (Tsai et al., 2016). In view of the above analysis, we propose the following hypothesis:

H1: Subjective wellbeing has a significant and positive effect on the mental health of college students.
H2: Physical exercise has a significant and positive effect on the mental health of college students.
H3: Positive psychological intervention have a significant and salutary outcome on college students' mental health.

### 2.2 Positive heart interventions and behavioral manifestations

Subjective wellbeing is a person's unique subjective evaluation of life, work and social relationships based on his or her own subjective criteria, marked by subjectivity and variability, wholeness and stability (Diener, 2000). The level of subjective wellbeing in a way shows the individual's mental health indicators, and strengthening the learning of subjective wellbeing has an important value and significance in regulating the mental health and practice behavior of college students (Yao et al., 2025). Adolescents‘ subjective wellbeing helps to stimulate their own heart potential, provide strong motivation for learning and development, regulate their behavior in practice, achieve satisfactory results, and upgrade the quality of living circumstances (Wu et al., 2023). Existing research suggests that having the capacity for subjective wellbeing is an important prerequisite for improved academic performance (Ansari and Stock, 2010), and that the significance of subjective wellbeing transcends the boundaries of the individual to promote positive and prosperous school cultures (Travia et al., 2022), enhance students' skills in communicating and interacting with others (Cobo-Rendón et al., 2020), thus helping students to act in a manner that tallies with the desired goals expected of them.

As the field of sport psychology continues to develop, participation in physical activity can regulate emotions, enrich the cognitive horizons of individuals, and also reduce the risk of developing mental health problems (Mahindru et al., 2023). Physical exercise refers to physical activities that are carried out by an individual according to a predefined program, following a fixed cycle and repetition, based on the mutual adaptation of his/her own reality and the external environment (Mileva and Zaidell, 2022). Physical activity is a healthy lifestyle that relies on the individual's own and external forces to exercise the body, which not only contributes to the construction of mind and body, but also adjusts and regulates the individual's habits and behaviors (Wei, 2023). Active sports participation not only demonstrates an individual's perspective and behavior toward physical activity, but also plays an important role in physical health, mental health, and social flexibility (Teixeira et al., 2012). Moderate physical activity enhances the structure and function of the brain, improves the body's memory capacity, and enhances the level of communication and interpersonal interactions, which results in positive feedback on human behavior and performance (Valkenborghs et al., 2019). People who regularly engage in physical activity will demonstrate an extremely high level of self-control and will manage their personal resources effectively in order to achieve long-term goals, and in turn will exhibit goal-directed behavioral performance (Eysenck et al., 2007). Therefore, we propose the following hypothesis:

H4: Subjective wellbeing holds a considerable and positive effect on the behavioral performance of college students.
H5: Physical exercise displays a pronounced and positive effect on the behavioral performance of college students.
H6: Positive psychological intervention have a significant and salutary consequence on university students' behavioral performance.

### 2.3 The moderating role of teacher empathetic concerns

Empathy refers to an individual difference that enables one to understand others‘ perspectives and feelings, care for their emotions and thoughts, and view and handle problems from their perspective (Weisz and Cikara, 2021; Howe, 2012). Empathy mainly encompasses two aspects of factors: emotions and cognition (Mencl and May, 2009). Empathy is an important condition for interpersonal communication. This psychological state is also felt by others even when one is following one's own desires (Main et al., 2017). It can not only satisfy an individual's subjective wellbeing but also provide a firm grounding for the development of mental health. For athletes, in order to achieve excellent results, they will spend a lot of energy on physical training (Edison et al., 2021). Under the influence of empathy, it can help athletes recover their injured bodies in time, enhance their self-confidence, better engage in physical training, and maintain a positive and good mindset. Caring behaviors demonstrated by teachers through emotional, pedagogical, and moral aspects can help students reduce anxiety and improve academic performance (Sutton and Wheatley, 2003). As a key aspect of teaching performance, teachers' empathy focuses on helping teachers‘ teaching content meet students' needs (Meyers et al., 2019). Sympathetic teaching methods can activate the classroom atmosphere, enhance students‘ learning motivation, and thereby more effectively improve students' learning behaviors (Cai et al., 2022). In conclusion, we propose the following hypotheses:

H7: Teacher empathic concern definitely moderates the partnership between positive heart interventions and college students' mental health.
H8: Teacher empathic concern indubitably moderates the bond between positive mindfulness interventions and college students' behavioral performance.

### 2.4 Research objective

**Figure 1:**
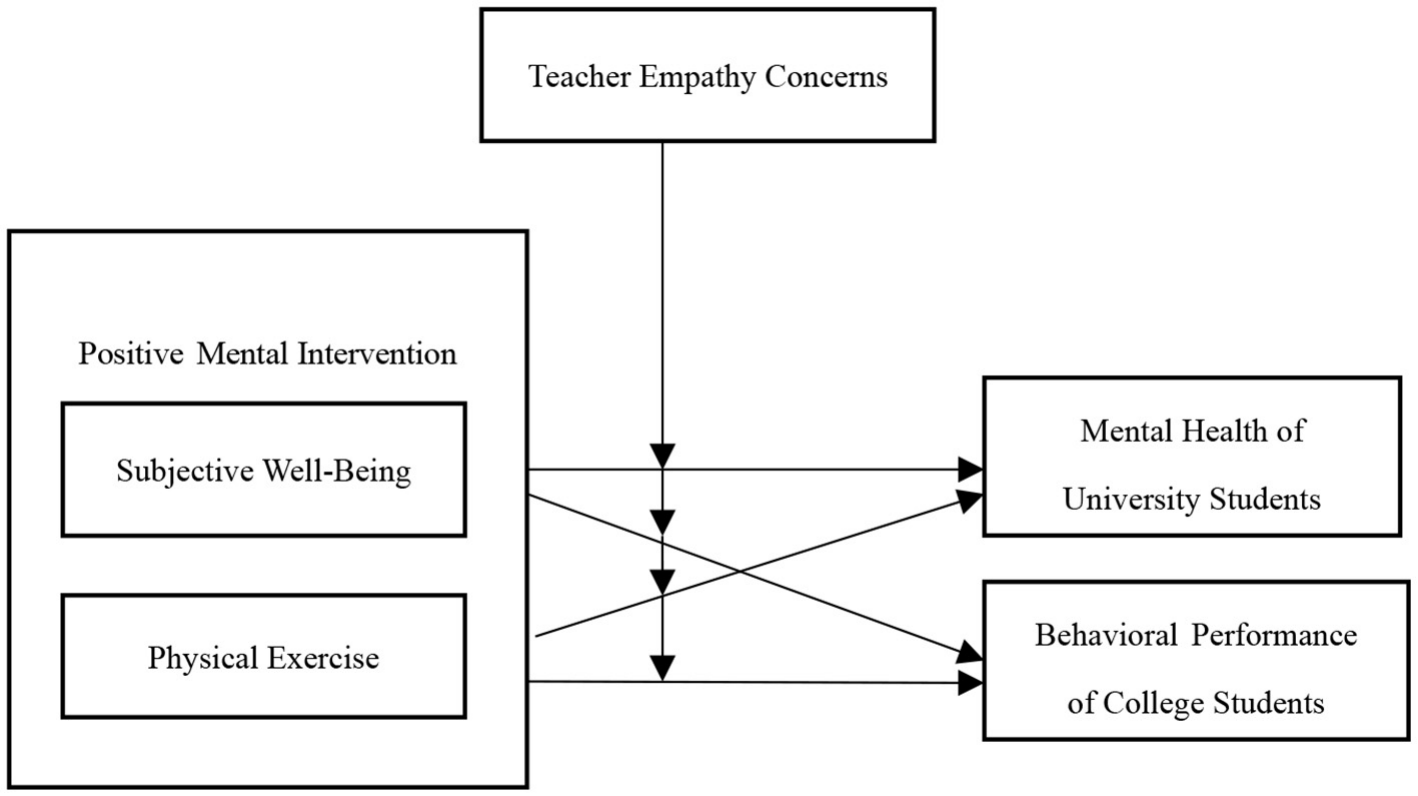
A Conceptual model of the relationship between positive mental interventions, subjective wellbeing, physical exercise, teacher empathic concerns, and college students' mental health and behavioral performance.

Numerous substantial pieces of evidence indicate that there is a significant relationship between positive heart interventions, subjective wellbeing, physical activity, empathetic attention, and college students‘ mental health and behavioral performance. We believe that integrating these factors into a model can more clearly demonstrate the developmental path of college students' mental health and behavioral performance, as shown in Figure 1.

## 3 Methods

### 3.1 Survey methodology

We used a random sampling method to collect data by conducting a questionnaire survey of colleges and universities in Anhui Province, China. We selected colleges and universities in 8 cities in Anhui Province according to the principle of random sampling. The selection includes colleges and universities in 1 capital city and 7 prefecture-level cities in Anhui Province. The questionnaire research was conducted through the Questionnaire Star platform from early March 2025 to late May 2025, and the questionnaire was collected in both online and field research, online through WeChat and email, and in the field through actual on-site interviews. Before the start of questionnaire collection, the full panel of participants were informed of the purpose of the study, procedures, voluntary participation, confidentiality, and consent was received from the participants, and all information was collected and used anonymously.

### 3.2 Participants

A total of 300 students participated in filling out the questionnaires and 282 were returned, excluding 22 invalid questionnaires that were filled out incorrectly, 260 valid questionnaires were finally collected and a valid response rate of 86.67% was obtained. Of these, 136 were male (52.31%) and 124 were female (47.69%). Of those who participated in the questionnaire, the distribution of participants was as follows: 83 (31.92%) were freshmen, 69 (26.54%) were sophomores, 63 (24.23%) were juniors, 23 (8.85%) were seniors, and 22 (8.46%) were researchers. Considering the data collection through online and on-site surveys, as well as the fact that the questionnaires that failed to be returned may have had an impact on this research, the application of the *t*-test showed that there was no significant difference between the 282 questionnaires that had been returned and the 18 questionnaires that had not been returned under the influence of the participants' gender and age, and that the *t*-test indicated that there was no unresponsiveness bias in the research questionnaires and that they could be suitable for further research. In addition, we conducted regression analyses for the online questionnaire and the on-site questionnaire separately, and the results of both were completely consistent, indicating that both the online and on-site questionnaires did not affect the conclusions of the study.

## 4 Measures

### 4.1 Subjective wellbeing

Referring to the question-item measurement scale proposed in the study of Diener et al. (1985) and others, the questionnaire was set up with five question items on a five-point Likert scale (“1-strongly disagree”, “5-strongly agree”), such as “I am satisfied with my studies and life” and “In many ways, my real life is in line with my ideal goals”. The higher the score, the better the quality of life of college students. In this study, the Cronbach coefficient of subjective wellbeing of college students was 0.924.

### 4.2 Physical exercise

Based on the Physical Activity Questionnaire Item Measurement Scale proposed by Sagatun et al. (2007) and others, the questionnaire was set up with five items on a five- scale Likert scale (questionnaire options coded 1-5), with questions such as, “What's the number of h you spend each week on participate in aerobic or sweaty exercise routines?”. The greater the numerical value of the grade, the more effective the bodily movements. The Cronbach coefficient for physical activity was 0.922.

### 4.3 Mental health of college students

Referring to the scales studied by Crawford and Henry (2003) and Gong et al. (2010), college students' mental health was set up with four questionnaire items covering three dimensions of depression, anxiety, and stress on a five-point attitude measurement scale (“1- utterly disagree,” “5- vehemently agree”). Greater scores indicate higher severity of heart health and significantly lower levels of mental health. The coefficient of mental health Cronbach of university students in this study was 0.925.

### 4.4 Behavioral manifestations of university students

College students' behavioral performance draws on the research scale of Kou and Wang (2003), with four questionnaire items, such as “I will feel very good when I can solace someone who is upset,” “I will almost never refuse help when it is sought by others,” “I will make charitable donations anonymously”. The questionnaire used a five-point Likert scale (1 = completely oppose, 5 = fully endorse). Superior scores reveal a better level of behavioral performance among college students. The Cronbach's coefficient for behavioral performance of college students in this study was 0.889.

### 4.5 Teacher empathetic concerns

Teacher empathy concerns were measured with reference to the research of Cai et al. (2022) regarding the teacher empathy measurement scale, which mainly covers the sentimental and neurocognitive dimensions of teacher empathy. In conjunction with the questionnaire research, we set up five questionnaire items (1 = absolutely disagree, 5 = unreservedly concur) by drawing on a five-point Likert scale. The Cronbach's index of this scale is 0.920, which implies that the teacher empathy concern measurement scale has high reliability.

### 4.6 Control variables

In an effort to ensure the stability of the findings and to exclude the influence of participants‘ own characteristics, we included participants' gender and age as control variables, which have been shown in previous studies to be closely related (Lueke and Assar, 2024) to the principal variables of this research (Li et al., 2025).

## 5 Results

### 5.1 Reliability and validity test

With a view to test the reliability and validity of the interview schedule, this paper used the statistical software SPSS27.0 to assessment the reliability and validity of the variables of subjective wellbeing, physical exercise, teacher empathy concern, college students' mental health and behavioral performance in order to validate the relationship between each variable. As can be seen in Table 1, the Cronbach's α coefficients of each research variable ranged from 0.889 to 0.925, the KMO values of the variables were distributed from 0.822 to 0.898, and the combined reliability (CR) ranged from 0.924 to 0.948, with the respective values higher than 0.7, which indicates that the variables have a high reliability condition. The factor loadings of each variable ranged from 0.835 to 0.933, reaching values higher than 0.5, which proves that the scale validity of the research variables is good. The AVE of each research variable ranged from 0.754 to 0.821, meeting the critical value higher than 0.5, indicating a good discriminant validity of respective research variable.

**Table 1:** Findings from the reliability and validity assessment of variables.

**Variate**	**Item**	**Load**	**Cronbach's α**	**KMO**	**AVE**	**CR**
SWB	SWB1	0.933	0.924	0.895	0.770	0.943
	SWB2	0.852				
	SWB3	0.856				
	SWB4	0.867				
	SWB5	0.879				
PE	PE1	0.922	0.922	0.898	0.764	0.941
	PE2	0.871				
	PE3	0.863				
	PE4	0.851				
	PE5	0.862				
MH	MH1	0.932	0.925	0.854	0.821	0.948
	MH2	0.904				
	MH3	0.894				
	MH4	0.894				
BP	BP1	0.923	0.889	0.822	0.754	0.924
	BP2	0.849				
	BP3	0.864				
	BP4	0.835				
TEC	TEC1	0.931	0.920	0.890	0.761	0.940
	TEC2	0.866				
	TEC3	0.854				
	TEC4	0.865				
	TEC5	0.843				

SWB, Subjective Wellbeing; PE, Physical Exercise; MH, Mental Health; BP, Behavioral Performance; TEC, Teacher Empathic Concern.

### 5.2 Descriptive statistics

For the relative reliability of the research conclusions, SPSS27.0 software was used to process the data for each variable as a means to preliminarily verify the research hypotheses of each variable. As demonstrated in Table 2, the regression coefficients of subjective wellbeing on university students' mental health and behavioral performance are (β = 0.429, *P* < 0.01) and (β = 0.448, *P* < 0.01), the regression coefficients of physical exercise on college students' mental health and behavioral performance are (β = 0.390, *P* < 0.01) and (β = 0.352, *P* < 0.01), and the regression coefficients of positive heart intervention on college students' The regression coefficients of mental health and behavioral performance were (β = 0.487, *P* < 0.01) and (β = 0.477, *P* < 0.01), respectively, and the above correlation coefficients were significant, which preliminarily verified the hypotheses put forward in this study.

**Table 2:** Table of correlation parameters of variables.

**Variate**	**1**	**2**	**3**	**4**	**5**	**6**	**7**	**8**
1	1.000							
2	0.008	1.000						
3	0.019	−0.044	1.000					
4	−0.011	−0.064	0.840^**^	1.000				
5	0.043	−0.010	0.840^**^	0.411^**^	1.000			
6	0.053	0.017	0.487^**^	0.429^**^	0.390^**^	1.000		
7	0.009	−0.070	0.477^**^	0.448^**^	0.352^**^	0.405^**^	1.000	
8	0.043	−0.012	0.482^**^	0.332^**^	0.477^**^	0.416^**^	0.438^**^	1.000

1: gender; 2: age; 3: positive heart interventions; 4: subjective wellbeing; 5: physical activity; 6: college students‘ mental health; 7: college students' behavioral performance; 8: teacher empathetic attention. ^**^P < 0.01.

As can be seen from Table 3, the absolute value of skewness is less than 3, and the absolute value of kurtosis is less than 10, indicating that the skewness and kurtosis of each measurement question item are in a reasonable range, and the data of this study is characterized by a positive-too distribution, which constructs a solid foundation for the next step of carrying out regression analysis.

**Table 3:** Descriptive statistical analysis of variables.

**Variate**	**Item**	**AV**	**SD**	**Skewness**	**Kurtosis**	**Minimum**	**Maximum**
SWB	SWB1	3.3769	1.41839	−0.435	−1.135	1	5
	SWB2	3.2615	1.13256	−0.303	−0.684	1	5
	SWB3	3.2385	1.07842	−0.320	−0.620	1	5
	SWB4	3.2346	1.13676	−0.296	−0.776	1	5
	SWB5	3.1154	1.05914	−0.272	−0.783	1	5
PE	PE1	3.4462	1.37861	−0.439	−1.112	1	5
	PE2	3.2346	1.11965	−0.158	−0.842	1	5
	PE3	3.1769	1.15224	−0.366	−0.791	1	5
	PE4	3.2115	1.09291	−0.393	−0.673	1	5
	PE5	3.1654	1.11827	−0.181	−0.800	1	5
	MH1	3.2385	1.44288	−0.292	−1.310	1	5
MH	MH2	3.0769	1.16324	−0.195	−0.877	1	5
	MH3	3.0769	1.13977	−0.341	−0.829	1	5
	MH4	3.0769	1.19920	−0.176	−0.970	1	5
BP	BP1	3.5192	1.36829	−0.597	−0.880	1	5
	BP2	3.2538	1.07848	−0.334	−0.728	1	5
	BP3	3.2000	1.13491	−0.464	−0.589	1	5
	BP4	3.2154	1.06532	−0.150	−0.790	1	5
TEC	TEC1	3.4231	1.41074	−0.466	−1.079	1	5
	TEC2	3.2192	1.17979	−0.277	−0.932	1	5
	TEC3	3.1462	1.16984	−0.287	−0.859	1	5
	TEC4	3.2654	1.10926	−0.352	−0.696	1	5
	TEC5	3.2115	1.06428	−0.335	−0.702	1	5

AV, Average Value; SD, Standard Deviation.

### 5.3 Testing and analysis of research hypotheses

As shown in Table 4, Model 1 and Model 3 symbolize the effects of control variables on the dependent variables, i.e., the regression analysis of gender and age on the heart health and behavioral performance of college students correspondingly. Model 2 and Model 4 denote the effects of independent variables on dependent variables, i.e., regression analysis of subjective wellbeing and fitness routine on college students‘ mental health and behavioral performance respectively. The statistics in Table 4 proves that the effect of control variables on the dependent variable is negligible, indicating that gender and age do not affect this study. Subjective wellbeing (β = 0.327, *P* < 0.001), and physical exercise (β = 0.254, *P* < 0.001) are considerably related to the mental health of college students, so hypotheses H1 and H2 are verified. Subjective wellbeing (β = 0.362, *P* < 0.001), and physical exercise (β = 0.203, *P* < 0.001), all have a significant effect with college students' behavioral performance, so hypotheses H4 and H5 are validated.

**Table 4:** Regression analysis of positive heart intervention on college students' mental health and behavioral performance.

**Variate**	**Mental health**		**Behavioral performance**	
	**Model 1**	**Model 2**	**Model 3**	**Model 4**
Constant	2.906	0.817	3.408	1.564
Gender	0.118	0.101	0.019	0.009
Age	0.019	0.046	−0.072	−0.046
Subjective wellbeing		0.327^***^		0.362^***^
Physical exercise		0.254^***^		0.203^***^
*R* ^2^	0.003	0.242	0.005	0.237
Adjusted *R*^2^	−0.005	0.230	−0.003	0.225
*F*	0.396	20.387^***^	0.637	19.786^***^

^***^P < 0.001.

The results of the regression analysis between positive heart intervention, teacher empathy concern, college students‘ mental health and behavioral performance are shown in Table 5, and the analysis of the moderating effect is mainly carried out through three steps. Model 5 and model 8 are the effects of control variables on the dependent variable, i.e., the role of gender and age on college students' mental health and behavioral performance, and the results proved that the effects of control variables on the dependent variable were negligible. Model 6 and model 9 are the outcomes of independent variables on dependent variables, i.e., the ramifications of positive psychological intervention on college students‘ mental health and behavioral performance, and the evidence demonstrates that positive heart exert a substantial influence on college students' mental health (β = 0.488, *P* < 0.001) and behavioral performance (β = 0.474, *P* < 0.001), which signifies that positive psychological intervention has a significant positive influence so hypotheses H3 and H6 are validated. Model 7 and Model 10 are the effects of moderating variables between independent variables and dependent variables, i.e., the effects of teacher empathic concern between positive heart intervention and college students‘ mental health and behavioral performance, and the results show that there is a significant effect of teacher empathic concern between positive psychological intervention and college students' mental health (β = 0.332, *P* < 0.001), and positive psychological intervention and behavioral performance (β = 0.223, *P* < 0.001), i.e., teacher empathic concern has a positive moderating effect on positive psychological intervention and college students‘ mental health, and behavioral performance, so hypotheses H7 and H8 are verified.

**Table 5:** Regression analysis of positive psychological intervention, teacher empathic concern and college students' mental health and behavioral performance.

**Variate**	**Mental health**			**Behavioral performance**		
	**Model 5**	**Model 6**	**Model 7**	**Model 8**	**Model 9**	**Model 10**
Constant	2.906	0.829	0.785	3.408	1.586	1.419
Gender	0.118	0.097	0.050	0.019	−5.212	−0.036
Age	0.019	0.044	0.033	−0.072	−0.051	−0.058
Positive psychological intervention		0.488^***^	0.261^***^		0.474^***^	0.266^***^
Teacher empathy concern			0.253^***^			0.286^***^
TEC × PPI			0.332^***^			0.223^***^
*R* ^2^	0.003	0.241	0.382	0.005	0.229	0.331
Adjusted *R*^2^	−0.005	0.232	0.370	−0.003	0.220	0.318
*F*	0.396	27.051^***^	31.371^***^	0.637	25.408^***^	25.156^***^

^***^P < 0.001.

**Figure 2:**
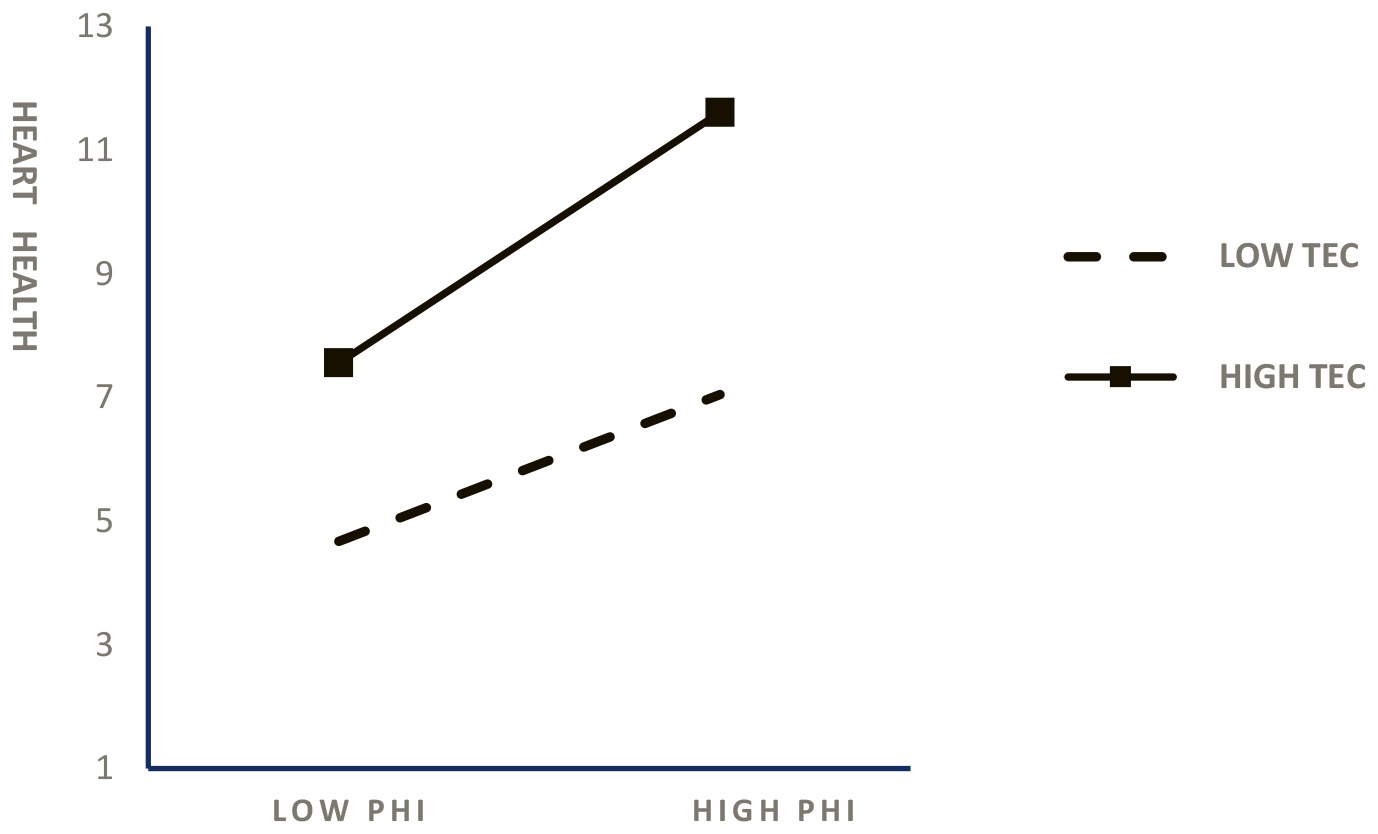
Moderating effects of teacher empathetic concerns on the link between positive heart interventions and college students' mental health.

**Figure 3:**
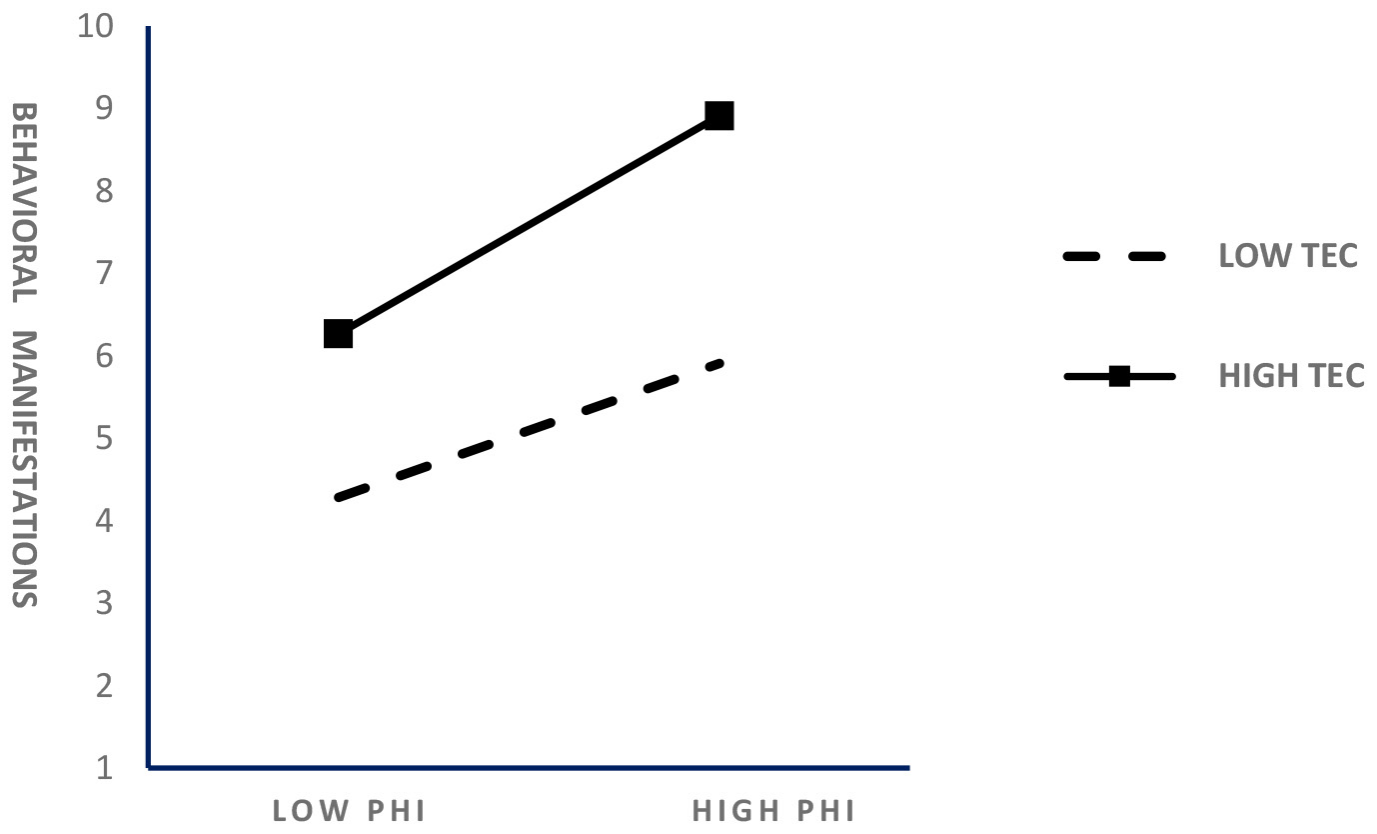
Moderating effect of teacher empathic concerns on the relationship between positive heart intervention and college students' behavioral performance.

In order to visualize the moderating effect, the moderating force diagrams of teacher empathic concern in the correlation between positive heart interventions and college students' mental health and behavioral performance are given, as shown in Figures 2, 3.

Based on the empirical analysis of the above study, the results of testing the detailed hypotheses are shown in Table 6.

**Table 6:** Results of testing each hypothesis.

**Hypothesis**	**Causal path**	**Coefficient**	**Significance**	**Result**
H1	SWB → MH	0.327	^***^	Supported
H2	PE → MH	0.254	^***^	Supported
H3	PPI → MH	0.488	^***^	Supported
H4	SWB → BP	0.362	^***^	Supported
H5	PE → BP	0.203	^***^	Supported
H6	PPI → BP	0.474	^***^	Supported
H7	TEC × PPI → MH	0.332	^***^	Supported
H8	TEC × PPI → BP	0.223	^***^	Supported

^***^P < 0.001.

## 6 Discussion

### 6.1 Subjective wellbeing

The present study proved that there is a significant positive correlation between subjective wellbeing on mental health and behavioral outcomes of university students. Our discoveries confirm previous research that adolescents‘ subjective wellbeing is receiving increasing attention (Alsarrani et al., 2022), and that subjective wellbeing can provide a more exhaustive mental standard of an individual's quality of life, which more accurately reflects an individual's social attributes and adaptive capacity. Adolescents tend to have lower levels of subjective wellbeing, which makes it important to emphasize and enhance adolescents' mental health during this period (Patton et al., 2016). In campus learning, students with a subjective sense of wellbeing are able to correctly grasp the feelings before, during, and after learning, and enjoy the achievements and happiness, thus maintaining a healthy state of heart (Gan and Lai, 2010). People with subjective wellbeing can better cope with external pressures, they are more capable of making themselves happy, more confident in their future development, and always maintain a better level of mental health (Diener and Seligman, 2004). A high level of subjective wellbeing not only promotes physical health and good social relationships, but also reduces the distress caused by depression and anxiety so that one's daily behavioral performance is normal (Burns et al., 2011).

### 6.2 Physical exercise

Our study confirms the contribution of physical activity to the mental health and behavioral performance of college students. Consistent with previous studies, insufficient exercise is a crucial factor in inducing mental health problems in college students, and long-term sedentary behavior can lead to excessive adiposity, visual impairment, and then severe heart problems (Brown et al., 2024). In the realm of exercise psychology, the relationship between physical exercise, physical health, and mental health is becoming increasingly close (Yang, 2016). Effective participation in physical activity can enhance an individual's sense of belonging, strengthen heart resilience, and improve interpersonal relationships (Yan et al., 2020). From a health promotion theoretical perspective, increased physical activity not only diminishes the incidence of disease, but also mitigates the stress of school and work and promotes an individual's heart health (Kim and Yoo, 2024). Physical activity provides an arena for adolescents to present themselves positively, which not only enables students to gain self-confidence and a sense of accomplishment, but also enables them to have a more conscious understanding of themselves and to align their behavioral performance with long-term plans (Meyer et al., 2021).

In conclusion, our investigation highlights that, in the first instance, college students who master subjective wellbeing will show a positive state toward learning and life, maintain a good level of mental health, and be able to regulate their behavioral performance at all times. On the other hand, regular physical exercise can also reduce the heart pressure of college students, less anxiety and depression, and in terms of psychology, it will help college students' mental health and behavioral performance significantly.

### 6.3 The moderating role of teacher empathetic attention

Our study elucidated the moderating role of faculty empathy concerns between positive heart interventions and college students‘ mental health and behavioral performance. Consistent with previous research, emotions and cognitions exhibited by empathy are important components of healthy living (Mencl and May, 2009), and empathy is also a positive factor for improved interpersonal relationships (Main et al., 2017). Our study can demonstrate through a questionnaire survey of college students that faculty empathy concerns can balance the relationship between positive heart interventions and college students' mental health and behavioral performance. Specifically, college students are at a stage that coincides with a critical period of becoming a mature person, facing anxiety about academics, social relationships, and the future, and will be in desperate need of positive heart interventions to help college students reduce their heart burdens. Teachers, as key players in the campus environment, are very important to students, and their empathetic attention, sympathy, concern, and help for students will more effectively play the role of positive heart interventions to maintain the level of mental health and behavioral performance of college students. Currently, college students lack sufficient knowledge about subjective wellbeing and physical activity, which can have an impact on the echelon of mental health and behavioral performance of college students. However, it has been recognized that subjective wellbeing is an effective mental intervention that will strengthen students‘ mental resilience and persevere in the pursuit of joy and happiness. Physical activity not only maintains a healthy body, but also releases academic stress and maintains physical and mental health in a variety of sports. In other words, teachers' empathetic attention will make positive heart interventions will more fully exert their effects on college students‘ mental health and behavioral performance. Our study further suggests that the cultivation of subjective wellbeing of and physical exercise can be incorporated into the philosophy of schooling and family education to improve college students' mental health and behavioral performance.

### 6.4 Limitations and future directions

Despite the fact that our study provides new empirical verification for the effects of subjective wellbeing, physical activity on college students‘ mental health and behavioral performance, and the positive moderating effect of teacher empathic concern, there are inevitably some limitations. First of all, the sample selection has limitations, this study only conducted research on college students in Anhui Province, and in the coming decades, we can broaden our scope the geographic area of the research, as well as the research object to cover primary education, junior secondary, and senior secondary. In addition, we only considered subjective wellbeing and physical exercise as positive heart interventions, and in the days ahead, we can further consider the sway of family environment, social support, self-control and other factors on college students' mental health and behavioral performance.

## 7 Conclusion

The outcomes of this investigation demonstrated a striking positive relationship between subjective wellbeing and physical activity on college students‘ mental health and behavioral performance, and also confirmed the positive moderating effect of teacher empathic concern. Specifically, subjective wellbeing positively enhances college students' mental health and behavioral performance, and physical activity positively contributes to college students‘ mental health and behavioral performance. It is worth noting that teacher empathic concern positively moderated the association between positive heart interventions and college students' mental health, and teacher empathic concern positively moderated the correlation between positive heart interventions and college students‘ behavioral performance. Although our study has some shortcomings, it helps to analyze the correlations between subjective wellbeing, physical activity, college students' mental health and behavioral performance, and teacher empathic concern. In addition, this study provides valuable suggestions for teachers, doctors, and parents to better safeguard the state of mental health of college students and to regulate their behavioral performance in line with the ideal goals they pursue.

## Data Availability

The original contributions presented in the study are included in the article/supplementary material, further inquiries can be directed to the corresponding author/s.
